# Theater-Based Participatory Action Research With Women Caring for Spouses With Dementia

**DOI:** 10.1093/geroni/igaf122.1809

**Published:** 2025-12-31

**Authors:** Meira Medina-Junge, Shoshi Keisari, Dovrat Harel

**Affiliations:** University of Haifa, Haifa, HaZafon, Israel; University of Haifa, Haifa, HaZafon, Israel; Tel-Hai College, Qiryat Shemona, HaZafon, Israel

## Abstract

Spouses caring for partners with dementia navigate complex challenges shaped by their marital life course. This study explored the experiences of older women caring for spouses with dementia and examined how Theater-Participatory Action Research (T-PAR) can serve as a tool for self-exploration and transformation. Over 10 sessions, seven female caregivers engaged in theatrical methods to examine the personal, familial, and social transitions they were undergoing. Guided recall interviews were conducted while reviewing video-recorded sessions, and the data were analyzed using an inductive reflexive thematic approach, integrating the Six-Keys model for analyzing dramatic scenes. Three main themes emerged: (a) redefining roles within the couple’s relationship, (b) redefining roles within their immediate environment, and (c) redefining roles in relation to healthcare institutions and authority figures. These themes were further captured through three experiential continua: (a) integration vs. splitting, (b) loneliness vs. shared experiences, and (c) suppression vs. activism. Findings highlight how theatrical methods provided a space for caregiver-women to explore shifting identities, rehearse coping strategies, and reimagine ways of relating to their partners and broader support systems. The discussion will focus on how drama-based participatory methods can empower spousal caregivers to navigate their evolving roles with agency and resilience.

